# Isothermal Amplification and Hypersensitive Fluorescence Dual-Enhancement Nucleic Acid Lateral Flow Assay for Rapid Detection of *Acinetobacter baumannii* and Its Drug Resistance

**DOI:** 10.3390/bios13100945

**Published:** 2023-10-23

**Authors:** Qian Wang, Shuai Zheng, Yong Liu, Chongwen Wang, Bing Gu, Long Zhang, Shu Wang

**Affiliations:** 1 Institutes of Physical Science and Information Technology, Anhui University, Hefei 230601, China; wangq0323@163.com; 2 Hefei Institute of Physical Science, Chinese Academy of Sciences, Hefei 230036, China; kapposnn@163.com (S.Z.); liuyong1s@163.com (Y.L.); wangchongwen1987@126.com (C.W.); 3 Department of Clinical Laboratory Medicine, Guangdong Provincial People’s Hospital (Guangdong Academy of Medical Sciences), Southern Medical University, Guangzhou 510000, China; 4 Wan Jiang New Industry Technology Development Center, Tongling 244000, China

**Keywords:** carbapenem-resistant *A. baumannii*, nucleic acid fluorescent lateral flow assay, loop-mediated isothermal amplification, multilayered quantum dot nanobead tag, simultaneous detection

## Abstract

*Acinetobacter baumannii* (*A. baumannii*) is among the main pathogens that cause nosocomial infections. The ability to rapidly and accurately detect *A. baumannii* and its drug resistance is essential for blocking secondary infections and guiding treatments. In this study, we reported a nucleic acid fluorescent lateral flow assay (NFLFA) to identify *A. baumannii* and carbapenem-resistant *A. baumannii* (CRAB) in a rapid and quantitative manner by integrating loop-mediated isothermal amplification (LAMP) and silica–based multilayered quantum dot nanobead tag (Si@MQB). First, a rapid LAMP system was established and optimised to support the effective amplification of two bacterial genes in 35 min. Then, the antibody-modified Si@MQB was introduced to capture the two kinds of amplified DNA sequences and simultaneously detect them on two test lines of a LFA strip, which greatly improved the detection sensitivity and stability of the commonly used AuNP-based nucleic acid LFA. With these strategies, the established LAMP-NFLFA achieved detection limits of 199 CFU/mL and 287 CFU/mL for the *Rec*A (house-keeping gene) and *bla*_OXA-23_ (drug resistance gene) genes, respectively, within 43 min. Furthermore, the assay exhibited good repeatability and specificity for detecting target pathogens in real complex specimens and environments; thus, the proposed assay undoubtedly provides a promising and low-cost tool for the on-site monitoring of nosocomial infections.

## 1. Introduction

*Acinetobacter baumannii* (*A. baumannii*), a prevalent opportunistic pathogen, is among the key pathogens that cause nosocomial infections (especially in intensive care units) [1,2,3]. *A. baumannii* tends to acquire extensive drug resistance or multidrug resistance genes at a frightening rate, especially resistance to carbapenems [4]. This microorganism has the remarkable ability to survive for a long time in hospitals, including on hospital beds, floors and instruments, and it can cause serious diseases [5,6,7]. Based on this, the World Health Organization (WHO) listed carbapenem-resistant *A. baumannii* (CRAB) as a key priority bacterium in 2019 [8]. Thus, researchers in clinical microbiology laboratories have recently developed many new approaches for CRAB detection, including DNA sequencing, matrix-assisted laser desorption ionisation time of flight mass spectrometry (MALDITOF-MS) and molecular level (PCR)-based methods [9,10,11]. Although these methods improve the specificity of pathogen analysis compared to traditional culture methods, they all rely on expensive instruments and professionally trained operators, which limits their utilisation in resource-limited environments. The lateral flow immunoassay (LFIA) has experienced significant advancements in the field of point-of-care testing and has emerged as a widely utilised diagnostic tool owing to its rapidity and practicality [12,13,14,15]. However, antibodies are the only recognition element in this method, and the detection process depends on the binding of bacterial surface antigens to specific antibodies, which faces challenges during the identification of drug-resistant strains [16]. Therefore, a rapid and accurate method for detecting CRAB is urgently needed and is essential for controlling hospital-acquired infections.

To address this problem, researchers have introduced nucleic acid amplification (such as polymerase chain reaction, recombinase polymerase amplification, and loop-mediated isothermal amplification) into LFA using nucleic acids as a recognition element to form the nucleic acid lateral flow assay (NALFA) [17,18,19]. After the universal recognition element at the end of the primer is modified, the target nucleic acid label is specifically recognised and captured by the capture element of the tags/T line on the test strip, resulting in a readable signal [20]. The NALFA-based method for rapid bacterial detection presents the following advantages: (i) It is a paper-based detection platform with several advantages, such as low development cost, easy production, and simple operation without the need for professional operators. (ii) In the NALFA assay platform, the reaction time of the immunoassay is short (<20 min), and multiple bacteria or their resistant types can be detected simultaneously by setting up multiple test lines on a single test paper. (iii) NALFA technology identifies bacterial drug resistance types at the genetic level via nucleic acid amplification without screening for specific antibodies.

Loop-mediated isothermal amplification (LAMP) is a novel, simple, and rapid nucleic acid amplification technique proposed by Notomi et al. [21]. Compared with other nucleic acid amplification methods, LAMP technology is among the most developed isothermal amplification techniques and exhibits several advantages, including simple operation and low cost [22]. Currently, the major methods for the detection of LAMP products are agarose gel electrophoresis, real-time PCR or turbidimetric assays [23,24,25]. However, most of these methods require specialised personnel, long assay times (>1 h) and expensive instrumentation. Considering the need for rapid detection of *A. baumannii* in clinical settings, we focus on the NALFA technology and apply it to the detection of LAMP products. Recently, researchers developed a LAMP combined with a colloidal gold-based lateral flow biosensor (LAMP-LFB) for the detection of *Staphylococcus aureus* (*S. aureus*) and methicillin-resistant *Staphylococcus aureus* (MRSA) within 80 min, which effectively enhances and optimises the detection process of the LAMP system [26]. However, the current NALFA method is limited in terms of its detection time, sensitivity and quantitative analysis capability, mainly owing to the reliance on colloidal gold colourimetric analysis.

Here, a loop-mediated isothermal amplification-based nucleic acid fluorescence lateral flow immunoassay system was proposed for the first time (LAMP-NFLFA) to identify *A. baumannii* and CRAB, in which silica sphere multiquantum dot nanobead shells (Si@MQB) were introduced as signal tags. Three distinct advantages of the LAMP-NFLFA detection system were clearly observed. First, the LAMP system was integrated with the NFLFA system, which can realise the quantitative detection of target primers within a short time. Secondly, the composite nanolabel was used in this study instead of conventional colloidal gold, and multilayer quantum dot nanobead (QB) shells externally adsorbed on silica spheres significantly enhanced the fluorescence intensity and increased the antibody binding sites, thereby improving the detection sensitivity. Third, a dual-channel detection platform for *A. baumannii* and CRAB was established to achieve the rapid detection of carbapenem resistance in a fluorescence testing system. The two bright red bands of the T-line were observed under UV lamp (365 nm) irradiation, and then the fluorescence signal was measured using a portable fluorescence reader for the quantitative analysis of *A. baumannii* and CRAB. Under optimal conditions, the sensitivity of the proposed LAMP-NFLFA system reached 199 CFU/mL (*Rec*A gene) and 287 CFU/mL (*bla*_OXA-23_ gene) for the detection of *A. baumannii* genes. In addition, the actual detection capability of the system was evaluated by testing sputum or urine samples from the clinic. The specificity, accuracy and reproducibility of the assay system indicated that the LAMP-NFLFA system presented significant potential in POCT.

## 2. Materials and Methods

### 2.1. Reagents and Materials

The instrumentation used for the study is described in the Supplementary Materials S1. The bacterial DNA extraction kit, goat anti-mouse IgG antibody, rabbit anti-6-FAM polyclonal antibody (6-FAM), and anti-digoxin antibody used in this paper were purchased from Tiangen Biotech Co., Ltd. (Shanghai, China), BBI Sciences Corporation (Shanghai, China), Otwo Biotech (Shenzhen, China), and Abbkine (Wuhan, China), respectively. DNA marker 2000, isothermal amplification PCR mix, and bovine serum albumin (BSA) were provided by Sangong Biotech Co., Ltd. (Shanghai, China). SYTO9, streptavidin (SA) and polyethylenimine (PEI), MES monohydrate (MES), N(3-dimethylaminopropyl)-N’-ethylcarbodiimide hydrochloride (EDC), N-hydroxysuccinimide (NHS), tetraethoxysilane (TEOS) and Tween-20 used in the synthesis of materials were obtained from Sigma-Aldrich (St. Louis, MO, USA). CdSe/ZnS-MPA QDs were ordered from Mesolight Inc. (Suzhou, China). The nitrocellulose membrane utilised in the immunochromatographic strips was procured from Sartorius (Madrid, Spain), while the remaining materials were sourced from Jieyi Biotechnology Co. (Shenzhen, China).

### 2.2. Primer Design

The *Rec*A (GenBank ID: LC014676) gene was used to design universal LAMP primers for the detection of *A. baumannii*, and carbapenem resistance was detected by using LAMP primers designed for the *bla*_OXA-23_ gene (GenBank ID: KP203815). The primer sets, which consisted of outer primers F3 and B3, inner primers FIP and BIP, and loop primers LF and LB, were designed using LAMP-specific software (PrimerExplore V). The 5′-end of FIP for the *Rec*A and *bla*_OXA-23_ genes were labelled with digoxin and biotin, respectively, while both 5′-ends of BIP were labelled with 6-FAM. The designed primers were validated for specificity by comparing them with BLAST sequences. The primer sequences, which are provided in the Supplementary Materials’ Table S1, were synthesised, purified and modified by Sangong Biotech Co., Ltd. (Shanghai, China).

### 2.3. LAMP Reaction

The LAMP reaction for CRAB detection was performed in accordance with the manufacturer’s instructions. Briefly, the LAMP assay was performed using a 25 μL reaction mixture consisting of 12.5 μL of 2× Lamp Master Mix, 0.5 μL of DNA Polymerase, 7 μL of primer mixture, 2 μL of DNA template and 3 μL of sterilised ddH_2_O. The detection system was performed with different concentrations of internal primers to obtain the highest detection efficiency of the amplified products on NFLFA (i.e., FIP/BIP: 2 μM, 4 μM, 6 μM, 8 μM, 10 μM). The amplification system was incubated at a constant temperature of 65 °C for 30 min in a water bath, followed by the reaction at 80 °C for 5 min to inactivate the enzyme.

### 2.4. Preparation of Si@MQB

**Figure 1:**
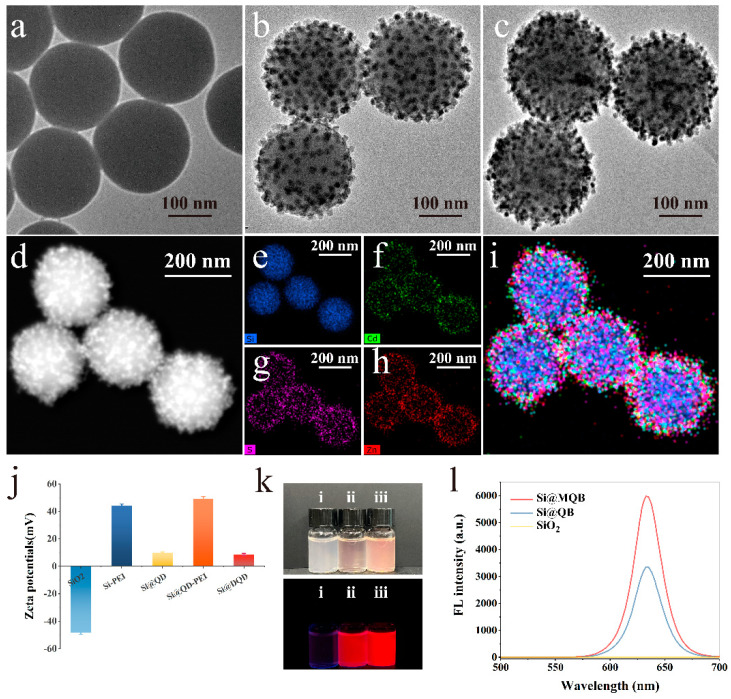
Characterisation of Si@MQB nanocomposites. TEM images of (**a**) SiO_2_, (**b**) Si@QB, and (**c**) Si@MQB. (**d**–**i**) Distribution of element mapping images for Si@MQB, Si (blue), Cd (green) and Zn (red). (**j**) Zeta potentials of the nanocomposites from each stage. (**k**) Images of (i) SiO_2_, (ii) Si@QB, and (iii) Si@MQB under daylight and UV light. (**l**) Fluorescence spectra of SiO_2_, Si@QB, and Si@MQB nanocomposites under UV light.

As illustrated in Figure 1a, the high-performance nanobeads were composed of a SiO_2_ [27] nanobead core (~180 nm) and a dual shell composed of QDs, which had adsorbed onto the surface layer by layer through electrostatic interactions. First, 1 mL of SiO_2_ nanoparticles was dispersed uniformly in 30 mL of deionised water. Subsequently, 1 mL of PEI solution (1 mg/mL) was added to the above solution and vigorously sonicated for 30 min to coat the surface of SiO_2_ nanobeads with PEI. Then, the synthesised Si@PEI nanoparticles were obtained through centrifugation at 4500 rpm for 6 min. After being washed twice with deionised water, the excess PEI was removed from the precipitate by purging. The resulting mixture was then resuspended in 30 mL of deionised water, and 0.2 mL of CdSe/ZnS-MPA QDs (10 nM) was added. After sonication for 45 min, the mixture was washed with deionised water and resuspended in another 30 mL of deionised water to form Si@QB. Si@MQB was prepared through the self-assembly of PEI onto Si@QB, followed by QD adsorption. After preparation, the final product was resuspended in 10 mL ethanol and stored away from light at 4 °C.

### 2.5. Preparation of Si@MQB-Anti-FAM Tags

High-performance tags were obtained by combining anti-FAM with Si@MQB via carbodiamine chemistry. First, ethanol in 1 mL of Si@MQBs was removed via centrifugation at 4000 rpm for 6 min. The resulting pellet was resuspended in a solution containing 0.5 mL of 0.1 M MES, followed by the addition of 10 μL of 0.1 M NHS and 50 μL of 0.1 M EDC for mixing. After reacting for 10 min, the Si@MQB spheres were separated from the solution and resuspended in 0.5 mL of a 10 mM PBS (pH 7.4) solution. The mixture was subsequently incubated with 3 μg of anti-FAM antibody for two hours. Then, 100 μL of 10% BSA was added, and shaking was continued for 1 h to block the unreacted carboxyl sites. Finally, the Si@MQB-anti-FAM spheres were collected and resuspended in 0.5 mL of 10 mM PBST (pH 7.4) for subsequent experiments.

### 2.6. Preparation of Samples

The *A. baumannii* strains used in this study, as well as *Pseudomonas aeruginosa* (*P. aeruginosa*), *Staphylococcus aureus* (*S. aureus*), *Enterococcus faecium* (*E. faecium*) and *Escherichia coli* (*E. coli*) for specific validation, were obtained from Guangdong Provincial People’s Hospital. The concentration and type of resistance of *A. baumannii* were determined using the classic plate counting method [28] (Figure S1a) and the Kirby–Bauer disc diffusion method [29], respectively. As shown in Figure S1b, the inhibition zone diameter of the tested strain was of less than 18 mm near the meropenem, indicating that it was resistant to carbapenem [30]. *A. baumannii* DNA (10^8^ CFU/mL) was extracted using a DNA kit and subsequently diluted with pure water to serve as a template for the LAMP reaction, ranging from 0 to 10^5^ CFU/mL. The mixture, consisting of 2 μL LAMP products, 80 μL running buffer (10 mM PBS containing 1% Tween 20), and 2 μL Si@MQB-anti-FAM tags, was then transferred to the NFLFA. The fluorescence intensity of the bands was measured using a portable fluorescence reader with 365 nm excitation after 6 min.

### 2.7. Fabrication of NFLFA

The NFLFA strip was composed of a base plate, sample pad, NC membrane, two test zones (T1 and T2), control line (C line) and absorption pad for the dual-channel identification of CRAB. A gold jet scribing instrument was used to spray the membrane with streptavidin (1 mg/mL), digoxin antibody (0.1 mg/mL) and goat anti-mouse IgG (0.8 mg/mL) as the test Lines T1, T2 and C, respectively. The final NFLFA test strips were cut to 3 mm and then stored in a desiccator.

### 2.8. Actual Sample Testing

The samples used in this study were swabbed from equipment and objects near patients and immersed in 2 mL of saline (sampling solution). Subsequently, different concentrations of CRAB were introduced into the sampling solution without the target strain for nucleic acid extraction, and the target gene was detected using the LAMP-NFLFA dual-channel detection system. Twelve clinical urine or sputum samples were collected from the Laboratory Department of Guangdong Provincial People’s Hospital. To identify the strains, genotypes and drug-resistant types, the samples first underwent MALDI-TOF-MS, PCR and AST. Then, the LAMP-NFLFA system proposed in this paper was applied. The samples with unknown concentrations were identified according to the fluorescence value and calibration curve.

## 3. Results and Discussion

### 3.1. Principle of LAMP-NFLFA

**Scheme 1:**
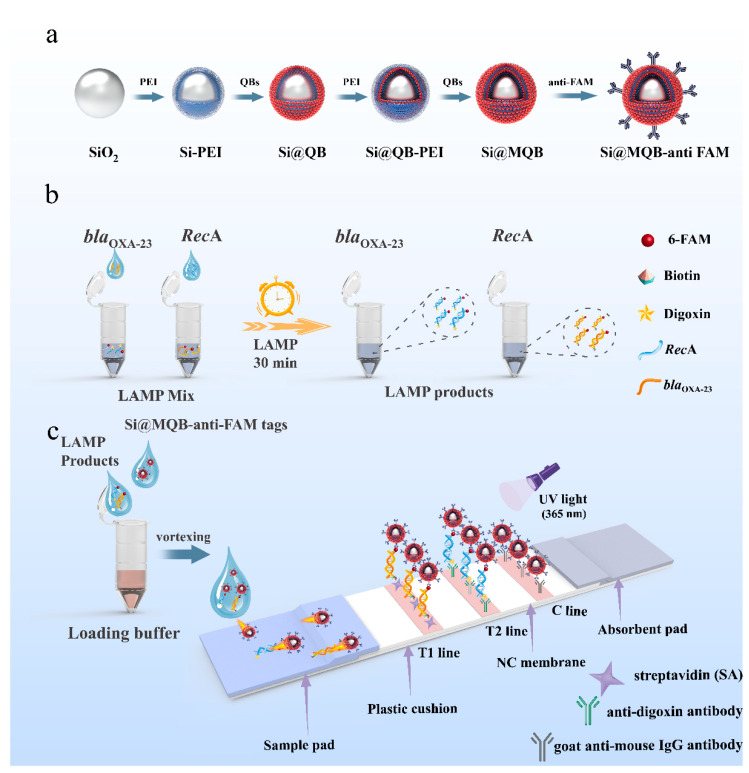
Schematic illustrations of the novel LAMP-NFLFA system for the detection of CRAB. (**a**) Preparation and modification process of Si@MQB-anti FAM tags. (**b**) The LAMP reaction process of amplification product binding to the tags. (**c**) Principle of LAMP-NFLFA assay for CRAB.

In this study, the sensitivity and detection stability of the NFLFA system were improved by introducing a silicon-core multilayer quantum dot shell (Si@MQB) as a detection tag to replace the conventional colloidal gold tag (Scheme 1a). The fluorescent tag exhibits the following characteristics: (i) SiO_2_ is selected as the carrier of composite nanotags, which can effectively improve the dispersion of signal tags to achieve high stability in the detection of complex samples. (ii) The added surface of the spherical nanotag allows thousands of CdSe/ZnS-MPA quantum dots to be loaded, which provides more information regarding the element binding sites and effectively improves the ability of the signal tag to capture the target sequence. (iii) The layer-by-layer assembly of the QD shell layer on SiO_2_ effectively enhances the loading rate of conventional quantum dot microspheres and improves the fluorescence intensity of signal tags. In addition, by detecting fluorescence signals, the NFLFA system could quantitatively detect analytes, further improving the detection accuracy.

The LAMP-NFLFA system mainly involves two steps, nucleic acid amplification (LAMP) and LFA (NFLFA), to achieve cascade signal amplification for the rapid detection of drug-resistant bacteria on-site with little sample usage. For the first step of the LAMP reaction, 6-FAM was added to the 5′-end of the BIP primers for *Rec*A and the *bla*_OXA-23_ gene, and biotin and digoxin were added to the 5′-end of the two primers FIP as tag markers (Scheme 1b). Through the specific recognition system (biotin/digoxin system and antigen/antibody system), the signal tags were effectively bound to the corresponding target nucleic acids, laying the foundation for the NFLFA detection system later. Subsequently, the LAMP reaction mixture remained in a constant temperature water bath at 60–65 °C for 30 min. Notably, the LAMP reaction was performed at a constant temperature, which allowed the basic thermostat to be sufficient.

During the NFLFA system assay, the amplified LAMP product in the loading solution can be specifically captured by the Si@MQB-anti-FAM tag to form the Si@MQB-DNA complex, which subsequently flows through the NFLEA strips that are intercepted by the recognition elements of the T lines (Scheme 1). As shown in Scheme 1c, detection elements such as anti-digoxin (T1) and streptavidin (T2) were immobilised onto the NFLFA strips and specifically bound to the target gene at the detection line (T1/T2) to generate fluorescent signals. A control region was also established using goat anti-mouse IgG to ensure the validity of the assay system. Next, a mixture consisting of Si@MQB tags, amplification products and loading buffer was added to the NFLFA test strip and migrated via capillary action. The Si@MQB-DNA complex was bound to the T line via an antigen-antibody specific reaction with the identification elements (digoxin antibody, streptavidin). The excess tags migrated and were subsequently captured by goat anti-mouse IgG, resulting in the appearance of a quality control line (C line). If the sample was the DNA from *A. baumannii*, a single fluorescent band was exclusively detected on the T line. If the bacterial DNA in the sample was CRAB, two distinct red bands could be observed under UV light. Through this design, the drug resistance typing of the target bacteria can be clearly identified by the number of bands. The fluorescence signal intensity was measured on the NFLFA using a portable fluorescence reader, resulting in a highly sensitive quantitative assay for the two specific genes of CRAB.

### 3.2. Characterisation of Si@MQB

The structures of SiO_2_ (~180 nm), Si@QB (~200 nm) and Si@MQB (~220 nm) were characterised via transmission electron microscopy (TEM). As shown in Figure 1a, the surface of the silica spheres with uniform particle size was smooth and subsequently became progressively rougher because the QBs were adsorbed on the surface with uniform and dense particles (Figure 1b,c). During the material synthesis process, the zeta potential also changed continuously due to the mutual attraction of positive and negative charges. As shown in Figure 1j, the zeta potentials of the five materials, including SiO_2_, Si-PEI, Si@QB, Si@QB-PEI and Si@MQB, were −48.3 mV, +44 mV, +9.8 mV, +49.3 mV and +8.5 mV, respectively. There was a significant increase in potential when the strongly positively charged PEI was adsorbed on SiO_2_, but the potential decreased again obviously as the nanomaterial was gradually wrapped with QBs; thus, electrostatic adsorption was effective for the layer-by-layer assembly of QBs. The analysis of the elemental composition and location distribution of Si@MQB was accomplished via EDS elemental mapping (Figure 1d–i). Si (blue) was densely wrapped by Cd (green) and Zn (red) elements, which presented a typical shell–core structure. Images showing SiO_2_, Si@QB and Si@MQB nanobeads under natural light and UV light are presented in Figure 1k, while their corresponding fluorescence spectra are displayed in Figure 1l. Specifically, the SiO_2_ spheres adsorbed with QBs showed a high emission peak at approximately 630 nm, while the pure SiO_2_ spheres exhibited no fluorescence signal. Meanwhile, the fluorescence signal intensity of composite nanolabels with different QB layers was measured. After adsorbing the second QB layer on the SiO_2_, the fluorescence intensity of the composite nanolabel increased significantly and the fluorescence performance of Si@MQB was improved by about two times compared to that of Si@QB. This evidence suggested that the high fluorescence signal emitted by Si@MQB originated from the PEI-mediated layer-by-layer self-assembly technique, which integrated multilayer QBs on a SiO_2_ core.

### 3.3. Detection Feasibility of the LAMP-NFLFA System

**Figure 2:**
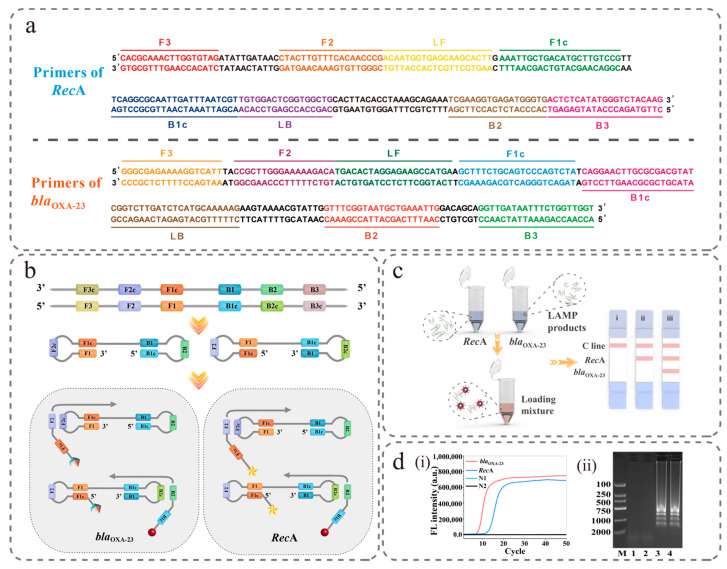
Schematic diagram for the construction of the LAMP-NFLFA system. (**a**) Primer and target binding sites for *Rec*A and *bla*_OXA-23_. (**b**) The LAMP amplification process. (**c**) Cross-validation analysis. (i) Negative control, (ii) containing only the *A. baumannii* DNA, (iii) containing the CRAB DNA. (**d**) Validation of amplification products. (**i**) RT-LAMP amplification curve, (**ii**) agarose gel electrophoresis bands, 1: nontarget control (*P. aeruginosa* DNA: 10^8^ CFU/mL), 2: no target control (NTC), 3–4: positive control (*A. baumannii* DNA:10^8^ CFU/mL), M: 2000 bp DNA marker.

In this paper, four sets of primers were designed for each of the two target genes of CRAB, and the best primer sets were chosen for subsequent experiments. And each set of primers consisted of outer primers (F3 and B3), inner primers (FIP and BIP) and loop primers (LF and LB) (Figure 2a, Supporting information Table S1). Among them, the addition of loop primers can significantly improve the amplification efficiency and reduce the reaction time. Notably, the design of the six primers used for the amplification reaction in the LAMP reaction ensures high specificity and exponential amplification of the reaction. Figure 2b demonstrates the principle of the LAMP method, in which a dumbbell structure is used as the starting sequence for LAMP amplification of the two target genes.

To validate the accuracy of the LAMP-NFLFA detection system, LAMP amplification products were assessed using three distinct methodologies. The results obtained by the RT-LAMP assay are shown in Figure 2d(i). The S-shaped curves in blue and red are the amplification curves (positive) of the *Rec*A gene and *bla*_OXA-23_, respectively, while the green and black straight lines are the negative controls for both genes. The results obtained via agarose gel electrophoresis are shown in Figure 2d(ii), in which a trapezoidal band (positive) was observed only in Lane 3 and Lane 4; in contrast, both Lane 1 (nontargeted control) and Lane 2 (negative control) showed negative results on agarose gel electrophoresis. Finally, the verified LAMP product was detected via NFLFA, and a specific red fluorescent signal was observed on the corresponding T line (Figure S2). In conclusion, the fluorescence signal of the T1/T2 line clearly shows an on/off pattern according to the different target nucleic acids present, which indicated that the detection of the target genes of CRAB (*Rec*A and *bla*_OXA-23_) via the LAMP-NFLFA system was feasible.

In addition, capture elements, including digoxin antibody and streptavidin, were accordingly assigned to the NC membrane to construct the T1/T2 line (T1 for *Rec*A gene and T2 for *bla*_OXA-23_ gene). The selectivity of the two T lines for target nucleic acids on NFLFA test strips was first assessed by detecting target nucleic acids amplified using different primers. As demonstrated in Figure 2c, two fluorescent bands appeared on the NFLFA when the sample was CRAB (Figure 2c(iii)), and only one was observed when the sample detected was non-carbapenem-resistant *A. baumannii* (Figure 2c(ii)). In contrast, no red fluorescent band was detected on the NFLFA when a negative control was added to the loading mixture (Figure 2c(i)). The results indicated that no cross-reactivity occurred between the detection of the two genes.

### 3.4. Optimisation of the LAMP-NFLFA System

**Figure 3:**
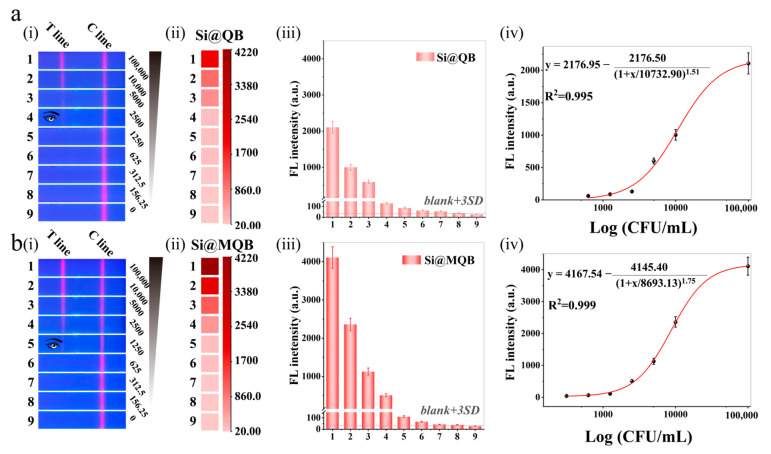
Optimisation of fluorescent tags. (**i**) Fluorescence images, (**ii**) heatmap results, (**iii**) fluorescence intensities, and (**iv**) calibration curves of (**a**) Si@QB-based and (**b**) Si@MQB-based LAMP-NFLFA for *bla*_OXA-23_ gene detection. The concentration of *bla*_OXA-23_ gene. 1: 100,000 CFU/mL, 2: 10,000 CFU/mL, 3: 5000 CFU/mL, 4: 2500 CFU/mL, 5: 1250 CFU/mL, 6: 625 CFU/mL, 7: 312.5 CFU/mL, 8: 156.25 CFU /mL, 9: 0 CFU/mL.

To improve the ability of the LAMP-NFLFA system to detect CRAB, we investigated the effect of self-assembling QD shells layer by layer on silicon spheres for fluorescent signal amplification. In theory, the fluorescence intensity produced by a tag is proportional to the number of QDs bound to it [31], which should improve the sensitivity of the LAMP-NFLFA system. Here, Si@QB and Si@MQB tags were synthesised and used to detect the same concentration of the *bla*_OXA-23_ gene (0–10^5^ CFU/mL) through the LAMP-NFLFA system. As shown in Figure 3a(i) and Figure 3b(i), the visual limits of detection (vLOD) [32] for Si@QB and Si@MQB were 2500 CFU/mL and 1250 CFU/mL, respectively. Obviously, the Si@MQB tag exhibited a more intense fluorescence signal on the T line compared to Si@QB. The fluorescence intensity of the red bands was then measured using a portable fluorescence reader under UV light at 365 nm. Figure 3a(i–iii),b(ii,iii) shows the changes in the fluorescence intensity for the two tags, Si@QB and Si@MQB, with the target nucleic acids being diluted. Next, the corresponding calibration curves were plotted based on the fluorescence signal measured on the T1/T2 line (Figure 3a(iv),3b(iv)). The LOD [33] of the assay system was determined according to the International Union of Pure and Applied Chemistry (IUPAC) protocol, which is the sum of the mean fluorescence intensity from multiple assays and the standard deviation of a 3-fold blank set. The LOD of Si@QBs was determined to be 664 CFU/mL, while that of Si@MQBs was 284 CFU/mL. The detection sensitivity of the LAMP-NFLFA system based on Si@MQB tags was more than two times higher than that of Si@QB, indicating that tags with multilayer QB shells could effectively improve the sensitivity of the LAMP-NFLFA detection system. In addition, Si@MQB tags are only a little bit more expensive than Si@QB tags after comparison.

In addition, to rapidly and accurately identify CRAB using the LAMP-NFLFA system, we optimised the LAMP reaction temperature, the concentration of primers, the volume of LAMP product added to the running buffer, the type of NC membrane, the composition of the running buffer and the reaction time. The highest signal-to-noise ratio (SNR) [34] was determined as the best reaction condition in this experiment, and the SNR was obtained by calculating the ratio of fluorescent signals for positive bands to negative bands.

Firstly, the LAMP reaction temperature was optimised. As shown in Figure S3, the earliest amplification curve appeared at 65 °C; therefore, it was considered that 65 °C was the optimal amplification temperature. The amplification efficiency for the target nucleic acid was highly dependent on the concentration of the primer, as a low concentration could lead to decreased efficiency. However, excessive primers in NFLFA might hinder the binding of the target nucleic acid to the recognition element immobilised on the NC membrane, leading to a reduction in the fluorescence signal intensity of the red band. Therefore, a concentration of 0.8 μM was chosen for both FIP and BIP in subsequent experiments (Figure S4). In addition, the volume of LAMP product added to the running buffer was optimised before the NFLFA reaction. As illustrated in Figure S5, the LAMP product with 2 μL exhibited optimal SNR. The detection efficiency of the target was significantly influenced by the type of NC membrane. In this study, NC membranes with different pore sizes, including 15 μm (CN95), 8 μm (CN140) and 17 μm (Vivid170), were investigated. As shown in Figure S6, the highest SNR was achieved when CRAB was detected with the CN140 membrane compared to other NC membranes. The running buffer used in NFLFA was then optimised because it greatly impacted the movement of the tag-nucleic acid complex on the NC membrane. As shown in Figure S7, PBST containing 1% Tween-20 maximised the SNR of the tested region. Finally, the optimal reaction time for NFLFA detection of CRAB was determined to be 8 min, allowing for shorter testing (Figure S8).

### 3.5. Construction of the LAMP-NFLFA Dual-Channel Method for the Detection of A. baumannii and CRAB

**Figure 4:**
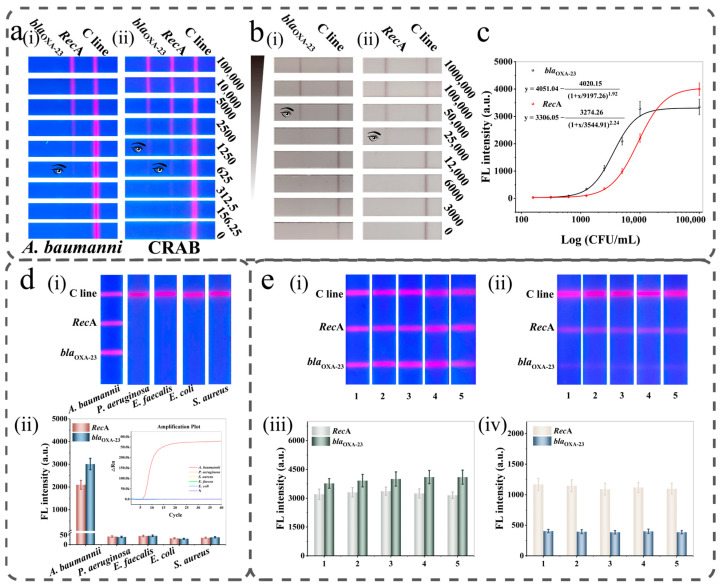
Sensitivity for *A. baumannii* and CRAB detection. (**a**) Fluorescence pictures. (**b**) AuNP-based NALFA pictures. (**c**) Corresponding calibration curves of LAMP-NFLFA for *Rec*A and *bla*_OXA-23_. (**d**) Specificity of CRAB. (**i**) Fluorescence images and (**ii**) corresponding fluorescence intensity of Si@MQB-based LAMP-NFLFA. (**e**) Reproducibility of LAMP-NFLFA for *Rec*A and *bla*_OXA-23_. Fluorescence images: (**i**) 10^5^ CFU/mL and (**ii**) 2500 CFU/mL, corresponding fluorescence intensity (**iii**) 10^5^ CFU/mL and (**iv**) 2500 CFU/mL.

Under optimised conditions, the sensitivity of the LAMP-NFLFA system was assessed using varying concentrations of DNA for CRAB (as described in the experimental section). The brightness of fluorescence on the two T lines gradually diminished with decreasing target nucleic acid concentration, and the vLOD of *Rec*A and *bla*_OXA-23_ genes were as low as 625 CFU/mL and 1250 CFU/mL, respectively (Figure 4a). The fluorescence intensity on the T-line was then detected by using a portable fluorescence reader, and calibration curves for both genes were constructed based on the measured fluorescence (Figure 4c). The formula used to calculate LOD from the calibration curve is shown in 3.4. Thus, the LOD of LAMP-NFLFA was determined to be 199 CFU/mL for *Rce*A and 287 CFU/mL for *bla*_OXA-23_. However, as a novel CRAB detection system, LAMP-NFLFA was compared to conventional colloidal gold-based NALFA [35] test strips at the same target gene concentration for its detection performance. As shown in Figure 3b, the vLOD of AuNP-based NALFA test strips for *Rec*A and *bla*_OXA-23_ were 2.5 × 10^4^ CFU/mL and 5 × 10^4^ CFU/mL, respectively. The sensitivity of LAMP-NFLFA for target gene detection was at least 125-fold higher than that of the AuNP-based NALFA method. In addition, we compared the detection method proposed in this paper for *A. baumannii* with other previously reported detection methods. As shown in Table 1, it is clear that the present study possesses the advantages of rapid detection and high sensitivity while being able to differentiate carbapenem-resistant *A. baumannii*. Therefore, the high-performance LAMP-NFLFA system designed in this study showed significant advantages in the detection of bacterial drug resistance in POCT.

We further tested other common pathogens, including *P. aeruginosa*, *E. faecalis*, *E. coli* and *S. aureus*, to verify the specificity of the prepared LAMP-NFLFA system. As shown in Figure 4d(i,ii), bright fluorescent bands were observed on the T1 and T2 lines for the CRAB group, while the other control samples showed no perceptible red fluorescent signal at the T zone. As predicted, the proposed assay specifically distinguishes CRAB from other common pathogens.

We further evaluated the reproducibility of the proposed LAMP-NFLFA system with five independent tests. Figure 4e(i–iv) shows the detection results of the LAMP-NFLFA system for DNA samples with high (10^5^ CFU/mL) and low (2500 CFU/mL) concentrations of CRAB. The fluorescence signal was subsequently measured, the fluorescence intensity of the NFLFA test strips within each group was fairly uniform, and the relative standard deviation (RSD) was less than 10%. Therefore, the LAMP-NFLFA system we proposed provided good reproducibility and specificity for the detection of CRAB.

### 3.6. Performance of Si@MQB-Based LAMP-NFLFA in Bacteria Detection

**Figure 5:**
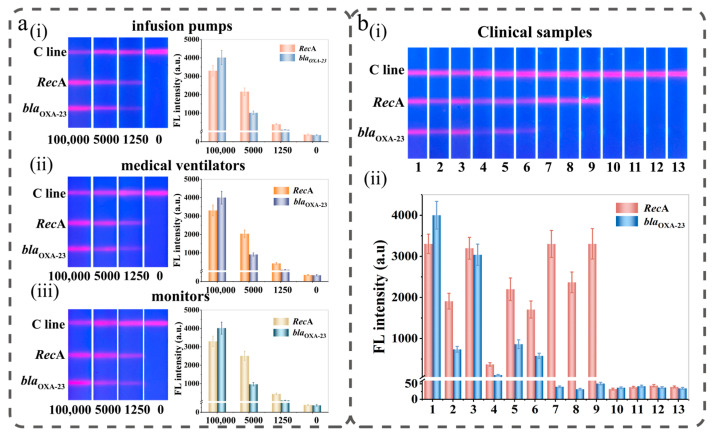
Performance of the LAMP-NFLFA system. (**a**) Sample solutions from (**i**) infusion pumps, (**ii**) medical ventilators and (**iii**) monitors. (**b**) Clinical samples 1–6: CRAB, 7–9: *A. baumannii*, 10–12: nontarget bacteria, 13: blank control.

To evaluate the effectiveness of the LAMP-NFLFA system for detecting real samples, simulated samples (sampling fluid from infusion pumps, medical ventilators and monitors) and actual samples (sputum or urine from the clinic) were used for validation. Pure cultures of CRAB (10^5^ CFU/mL, 5000 CFU/mL, 1250 CFU/mL) were first added to the sampling solution without the target strains, and then the results were analysed with the LAMP-NFLFA system constructed in this study. As shown in Figure 5a(i–iii), the fluorescence intensities of T1/T2 gradually weakened with decreasing target nucleic acid concentrations, showing a concentration-dependent trend. The difference in fluorescence intensity between the three groups of simulated samples at the same bacterial concentration may be caused by the different efficiency of nucleic acid extraction in each group. The mean recoveries of the LAMP-NFLFA system for CRAB after spiking were determined to be 83–119% according to the fluorescence signals obtained from each T-line (Table S2). These results showed that the LAMP-NFLFA system shows great potential for application in detecting CRAB on instruments.

With informed consent, the practical ability of the LAMP-NFLFA system to rapidly differentiate CRAB from other common pathogens was further validated using 12 clinical samples (sputum or urine) from Guangdong Provincial People’s Hospital. The results obtained for the LAMP-NFLFA system are shown in Figure 5b(i,ii) and were quantified based on the fluorescence signal measured using a portable fluorescence reader. As shown in Table S3, compared to the clinical identification results, the results obtained for *A. baumannii* and CRAB were 100% accurate. However, compared to the standard plate method, the quantification of clinical samples was performed with a correct rate of 84–116% using the LAMP-NFLFA system (Table S3). The above clinical results indicated that the assay system also shows strong applicability in clinical testing. Notably, the LAMP-NFLFA system developed in this study is a versatile assay that can be readily adapted for highly sensitive quantitative analysis of other pathogenic bacteria by utilising different primer sequences. With the addition of portable testing instruments, this testing system can be applied in various settings, such as hospitals and communities, to screen and monitor drug-resistant bacteria; thus, the system provides important technical support for the timely detection of drug-resistant bacteria.

## 4. Conclusions

In this paper, the isothermal amplification technique LAMP was successfully combined with the fluorescence detection platform NFLFA to design and test a method for the rapid detection of CRAB. Multilayer fluorescent labels (Si@MQB) were prepared using the PEI-mediated self-assembly method involving QBs and applied to the LAMP-NFLFA test system to replace the conventional colloidal gold labels. This method can realise the quantitative detection of *A. baumannii* and its carbapenem resistance genes by detecting the multilayer carboxylated QB signalling tags truncated on the corresponding T lines, and the detection sensitivity is as low as 199 CFU/mL and 287 CFU/mL, respectively, with high specificity and sensitivity. Moreover, CRAB can be discriminated under optimal conditions, the detection time is less than 43 min, and no complicated equipment or trained technicians are necessary. Most importantly, by combining different amplification primers, our LAMP-NFLFA system provides a universal LFA system that realises the rapid detection of different pathogens without the need for time-consuming antibody screening steps. We believe that the proposed LAMP-NFLFA can provide an efficient tool for the timely and accurate determination of pathogens and their drug resistance in clinical specimens and hospital environments. Though a UV light or fluorescence reader is required for this experiment, the cost is very low compared to that of instruments such as PCR and DNA sequencing. In the future, the cost can be further reduced by developing handheld instruments.

## Figures and Tables

**Table 1:** Comparison of the analytical properties of the developed assay with other methods for *A. baumannii*.

Detection Method	Differentiation of Drug Resistance	Detection Limit	Assay Time (min)	Reference
RT multiplex PCR	No	8333 CFU/mL	>60	[36]
Fluorescence	No	10^3^CFU/mL	40	[37]
Microfluidics	No	10^2^ CFU/mL	90	[38]
Microfluidics	No	10^4^ CFU/mL	30	[39]
LAMP	Yes	10 pg	60	[40]
LAMP	No	10^4^ CFU/mL	15	[41]
LAMP	Yes	10^4^ CFU/mL	−	[42]
Electrochemical Genosensor	No	10^3^ CFU/mL	−	[43]
LAMP-NFLFA	Yes	199 CFU/mL	43	This work

## Data Availability

Data will be made available on request.

## Supplementary Materials

The following supporting information can be downloaded at: https://www.mdpi.com/article/10.3390/bios13100945/s1.
