# Supplementary material for: Isothermal Amplification and Hypersensitive Fluorescence Dual-Enhancement Nucleic Acid Lateral Flow Assay for Rapid Detection of Acinetobacter baumannii and Its Drug Resistance
Source: Biosensors (Basel). 2023 Oct 23;13(10):945. doi: 10.3390/bios13100945 (PMC10605404; doi:10.3390/bios13100945)
Supplement: Supplementary file 1 [file biosensors-13-00945-s001.zip › biosensors-2643412-supplementary.pdf]

Supporting Information

Isothermal amplification and hypersensitive fluorescence dual-enhancement nucleic acid lateral flow assay for rapid detection of *Acinetobacter baumannii* and its drug resistance

Qian Wang[1,2,3], Shuai Zheng[2,3], Yong Liu[2,4], Chongwen Wang[2,3], Bing Gu[3,*], Long Zhang[2,*], Shu Wang[2,4,*]

[a] Institutes of Physical Science and Information Technology, Anhui University, Hefei 230601, China; wangq0323@163.com

[b] Hefei Institute of Physical Science, Chinese Academy of Sciences, Hefei 230036, China; kapposnn@163.com (S.Z.); liuyong1s@163.com (Y.L.); wangchongwen1987@126.com (C.W.)

[c] Department of Clinical Laboratory Medicine, Guangzhou, Guangdong Provincial People's Hospital (Guangdong Academy of Medical Sciences), Southern Medical University, Guangzhou, Guangdong 510000, China

[d] Wan Jiang new industry technology development center, Tongling 244000, PR China.

[*]Corresponding author

Email: gubing@gdph.org.cn (Bing Gu)

Email: zhanglong@aiofm.ac.cn (Long Zhang)

Email: wangshu87@163.com

wangshu@aiofm.ac.cn (Shu Wang)

S1. Instruments

The test strips were prepared by the QG001 high-speed continuous strip cutter and gold jet scribing instrument (Haining Wilfen Automation Equipment Co., Ltd., China). The fluorescence signals of the test strips were recorded with the -FIC-S1-Fluorescent Strip

Reader (Suzhou Hemi, China). Transmission electron microscopy (TEM) images were acquired for SiO_2, Si@OD, Si@DOD and CdSe/ZnS-MPA QDs using a Tecnai G2 F20 microscope operating at 200 kV (FEI Hong Kong Ltd., China). The fluorescence characteristics of the prepared nanocomposites were analyzed with a Fast Ocean Plus Act2 spectrometer (Chelsea, UK).

S2. Preparation of AuNPs tags

We first synthesized 40 nm Au nanoparticles (AuNPs) using the sodium citrate reduction method[1]. Next, we adjusted the pH of solution to 9 with 0.2 M K_2CO_3 and incubated them with 1 mL AuNPs for 15 minutes. Subsequently, 50 μL of BSA (10%) was introduced to block any unreacted sites on the surface of AuNPs. The AuNPs were isolated via centrifugation (4000 rpm, 6 min), and then resuspended in a storage solution consisting of 10 mM PB buffer (1% BSA, 0.1% PVP, 10% sucrose, and 0.05% Tween-20).

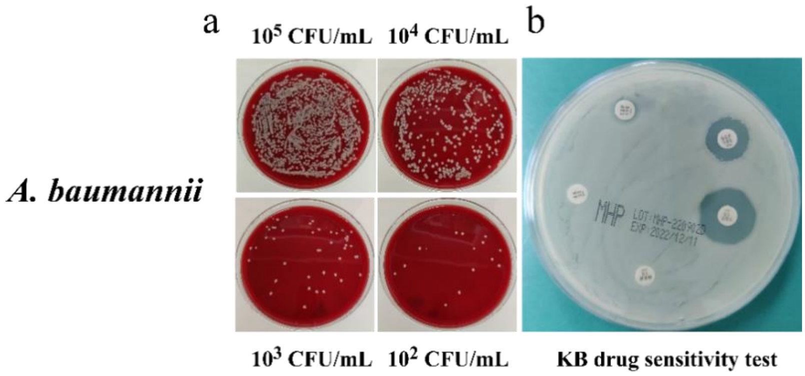

Fig. S1. Photographs of *A.baumannii* (a) colony growth on the blood agar plates (200 µL of the bacterial samples with different concentrations (10^5–10^2 CFU/mL) was coated on the blood agar plates), (b) KB drug sensitivity test.

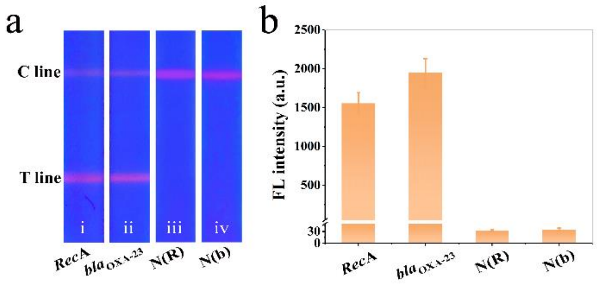

Fig. S2. LAMP products detection on NFLFA. (a) Fluorescence images. positive of (i) *Rec*A (ii) bla_{OXA-23}, negtive of (i) *Rec*A (ii) bla_{OXA-23}. (b) corresponding fluorescence intensities.

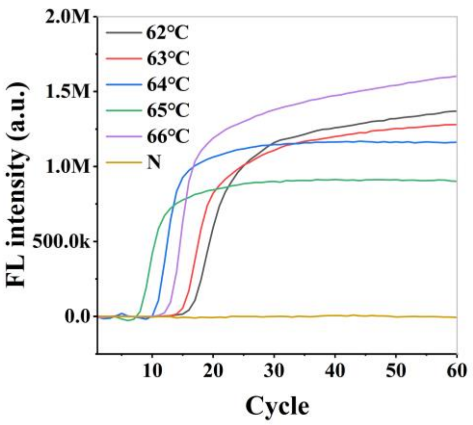

Figure S3 Optimization of the LAMP reaction temperature of *Rec*A

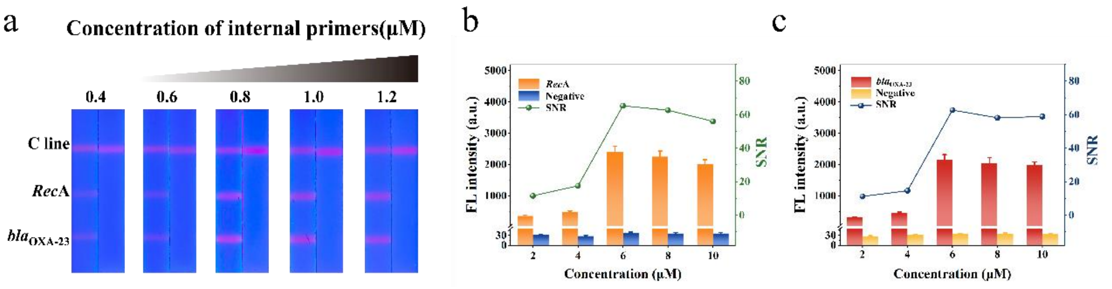

Fig. S4. Optimization of internal primers concentration for LAMP-NFLFA strip. (a) Fluorescence images and (b) corresponding fluorescence intensities for *Rec*A. (c) corresponding fluorescence intensities for *bla*$_{OXA-23}$. The error bars indicate standard deviations calculated from three measurements.

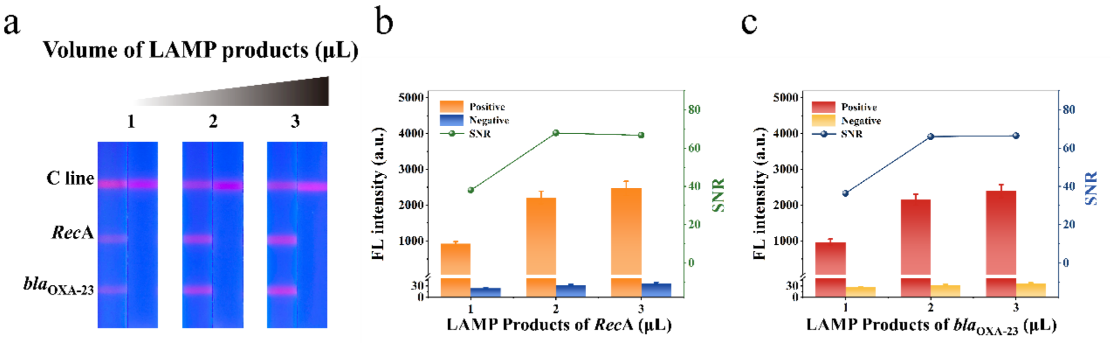

Fig. S5. Optimization of LAMP products volume for LAMP-NFLFA strip. (a) Fluorescence images and (b) corresponding fluorescence intensities for *Rec*A. (c) corresponding fluorescence intensities for *bla*$_{OXA-23}$. The error bars indicate standard deviations calculated from three measurements.

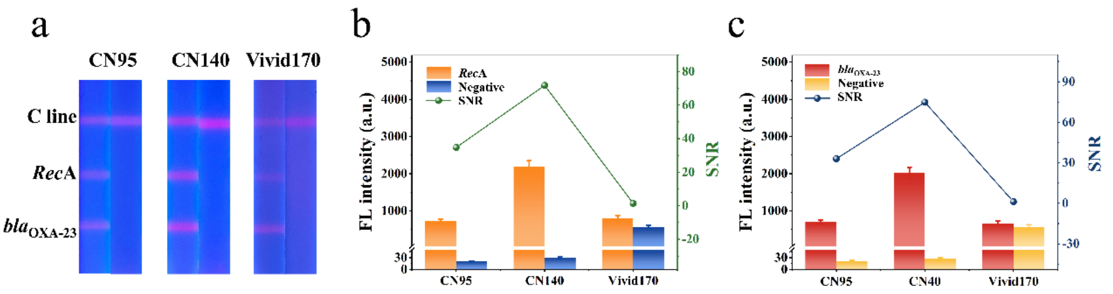

Fig. S6. Optimization of NC membrane for LAMP-NFLFA strip. (a) Fluorescence images and (b) corresponding fluorescence intensities for *Rec*A. (c) corresponding fluorescence intensities for *bla*$_{OXA-23}$. The error bars indicate standard deviations calculated from three measurements.

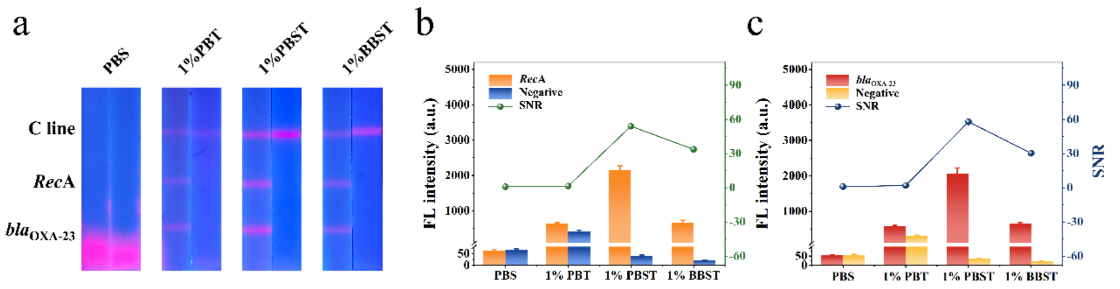

Fig. S7. Optimization of running buffer for LAMP-NFLFA strip. (a) Fluorescence images and (b) corresponding fluorescence intensities for *Rec*A. (c) corresponding fluorescence intensities for *bla*OXA-23. The error bars indicate standard deviations calculated from three measurements.

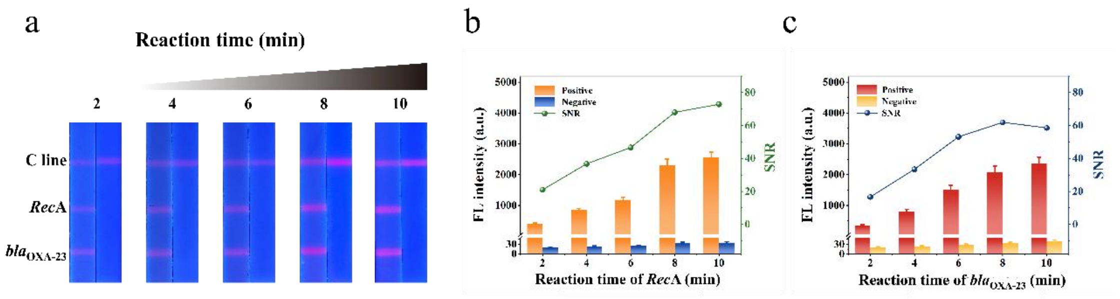

Figure S8. Optimization of the reaction time of the LAMP-NFLFA system. (a) Fluorescence images and (b) corresponding fluorescence intensities for *Rec*A. (c) corresponding fluorescence intensities for *bla*OXA-23. The error bars are derived from the standard deviations of three independent tests.

Table S1. Primers sequences used in this study.

Target gene	Primer	Sequence (5’-3’)	mark
*Rec*A	FIP	CGGACAAGCATGTCAGCAATTT-CTACTTGTTTCACAACCCG	5`-labeled Digoxin
	BIP	TCAGGCGCAATTGATTTAATCGT-CACCCATCTCACCTTCGA	5`-labeled 6-FAM
	LF	AAGTGCTTGCTCACCATTGT	
	LB	TGTGGACTCGGTGGCTG	
	F3	CACGCAAACTTGGTGTAG	
	B3	CTTGTAGACCCATATGAGAGT	
*bla*OXA-23	FIP	TAGACTGGGACTGCAGAAAGC-CCGCTTGGGAAAAAGACA	5`-labeled 5`Biotin
	BIP	CAGGAACTTGCGCGACGTAT-CAATTTCAGCATTACCGAAAC	5`-labeled 6-FAM
	LF	TCATGGCTTCTCCTAGTGTCA	
	LB	CGGTCTTGATCTCATGCAAAAAG	

	F3	GGGCGAGAAAAGGTCATT
	B3	ACCAACCAGAAATTATCAACC

Table S2. Recovery efficiency of *Rec*A and *bla*$_{\mathrm{OXA\text{-}23}}$ gene.

Sample	Spiked (CFU/mL)		Found (CFU/mL)		Recovery (%)		RSD (%, n=3)	
	*Rec*A	*bla*$_{\mathrm{OXA\text{-}23}}$	*Rec*A	*bla*$_{\mathrm{OXA\text{-}23}}$	*Rec*A	*bla*$_{\mathrm{OXA\text{-}23}}$	*Rec*A	*bla*$_{\mathrm{OXA\text{-}23}}$
Infusion pumps	10^5	10^5	9.57×10^4	1.08×10^5	95	108	8.8	9.7
	5×10^3	5×10^3	4.69×10^3	5.14×10^3	93	103	9.2	8.9
	1.25×10^3	1.25×10^3	1.40×10^3	1.12×10^3	112	90	10.1	11
Medical ventilators	10^5	10^5	8.84×10^4	9.13×10^4	88	91	8.9	8.7
	5×10^3	5×10^3	4.36×10^3	4.77×10^3	87	95	9.5	9.3
	1.25×10^3	1.25×10^3	1.46×10^3	1.06×10^3	117	85	10.7	13.8
Monitors	10^5	10^5	1.12×10^5	1.13×10^5	112	113	8.5	8.2
	5×10^3	5×10^3	5.90×10^3	4.94×10^3	118	98	9.8	9.4
	1.25×10^3	1.25×10^3	1.49×10^3	1.04×10^3	119	83	10.4	10.5

Table S3. Details of bacterial strains from clinical.

Serial No.	MALDITOF-MS	Genotypic		AST results	
	bacterial strains	*Rec*A	*bla*$_{\mathrm{OXA\text{-}23}}$	Meropenem	Imipenem
1	*A. baumannii*	+	+	R	R
2	*A. baumannii*	+	+	S	R
3	*A. baumannii*	+	+	R	R
4	*A. baumannii*	+	+	R	S
5	*A. baumannii*	+	+	R	S
6	*A. baumannii*	+	+	R	R
7	*A. baumannii*	+	-	S	S
8	*A. baumannii*	+	-	S	S
9	*A. baumannii*	+	-	S	S
10	*E. faecalis*	*Non-Acinetobacter*			
11	*P. aeruginosa*				
12	*S. aureus*				

AST, antimicrobial sensitivity testing；R, resistant; S, sensitive

Table S4. Recovery efficiency of *Rec*A and *bla*$_{\mathrm{OXA\text{-}23}}$ gene of clinical samples.

Sample	Plate (CFU/mL)		Found (CFU/mL)		Recovery (%)		RSD (%, n=3)	
	*Rec*A	*bla*$_{\mathrm{OXA\text{-}23}}$	*Rec*A	*bla*$_{\mathrm{OXA\text{-}23}}$	*Rec*A	*bla*$_{\mathrm{OXA\text{-}23}}$	*Rec*A	*bla*$_{\mathrm{OXA\text{-}23}}$
1	8.50×10^4	8.50×10^4	9.00×10^4	9.00×10^4	106	106	7.1	8.4
2	4.80×10^3	4.80×10^3	4.05×10^3	4.12×10^3	84	86	10.0	9.5
3	1.50×10^4	1.50×10^4	1.60×10^4	1.62×10^4	106	108	8.3	8.5
4	1.60×10^3	1.60×10^3	1.35×10^3	1.20×10^3	84	75	11.0	13.6

5	5.60×10^3	5.60×10^3	4.79×10^3	4.57×10^3	85	81	12.4	12.4
6	3.80×10^3	3.80×10^3	3.62×10^3	3.52×10^3	95	92	12.2	10.8
7	8.00×10^4	-	9.00×10^4	-	105	-	9.9	-
8	5.00×10^3	-	4.05×10^3	-	107	-	10.7	-
9	1.30×10^5	-	1.60×10^4	-	116	-	11.2	-
10								
11					*Non-Acinetobacter*			
12								
